# Early Effects of Repetitive Transcranial Magnetic Stimulation Combined With Sertraline in Adolescents With First-Episode Major Depressive Disorder

**DOI:** 10.3389/fpsyt.2022.853961

**Published:** 2022-07-19

**Authors:** Haisi Chen, Xiwen Hu, Jingfang Gao, Huan Han, Xiaole Wang, Chuang Xue

**Affiliations:** ^1^Affiliated Mental Health Center and Hangzhou Seventh People’s Hospital, Zhejiang University School of Medicine, Hangzhou, China; ^2^The First School of Clinical Medicine, Zhejiang Chinese Medical University, Hangzhou, China; ^3^The First Affiliated Hospital of Zhejiang Chinese Medical University (Zhejiang Provincial Hospital of Traditional Chinese Medicine), Hangzhou, China

**Keywords:** transcranial magnetic stimulation, adolescent depression, cold executive function, THINC-it, IVA-CPT

## Abstract

**Background:**

Adolescence is a period of high incidence for depression. However, there is a limited treatment option for the adolescent depression. For treatment-resistant major depressive disorder, HF-rTMS of the left dorsolateral prefrontal cortex (DLPFC) appears therapeutically effective. The aim of the study is to explore the early effects of repetitive transcranial magnetic stimulation in combination with sertraline in adolescents with first-episode major depressive disorder.

**Methods:**

A total of 100 teenage patients with first-episode depression were randomly divided into the study groups. Both groups were treated with sertraline. In addition, the study group was treated with ten sessions of add-on rTMS. The control group was given sertraline only. The depressive symptom and cognitive function were assessed by the Hamilton depression rating scale 17 version (HAMD-17), Children’s Depression Rating Scale-Revised (CDRS-R), Integrated visual and auditory continuous performance test (IVA-CPT), and THINC-it.

**Results:**

The number of early improvers after 2 weeks of treatment in the study group was statistically significant higher compared to the control group (95.83% vs 73.47%, χ^2^ = 9.277, *P* = 0.002). There was significant difference observed in responder rates (62.50% vs. 28.57%, χ^2^ = 11.262, *P* = 0.001) or in remission rates (31.25% vs. 6.12%, χ^2^ = 10.130, *P* = 0.001) between the two groups at 4 weeks. The score of HAMD-17 and CDRS-R in the study group were significantly lower than the control group (F_group_ = 12.91 vs 10.21, *P* < 0.05). Attention Quotient (listening, visual and full-scale) attention quotient of IVA-CPT in the study group were higher than those in the control group after treatment, and the differences were statistically significant(*P* < 0.05). The study group showed higher score in Spotter than the control group after treatment (*P* < 0.05).

**Discussion:**

This is the most extensive blinded, randomized clinical study to date examining the efficacy of 10-Hz add-on rTMS for first-onset adolescent depression. Our results support that add-on rTMS accelerates the efficacy of the antidepressants, improving the depressive symptoms and cold cognitive function in first-episode adolescent depression.

**Clinical Trial Registration:**

[www.ClinicalTrials.gov], identifier [ChiCTR2100048534].

## Background

Adolescence is a risk period for the onset of depression and the point prevalence for depression are estimated to be 8% (1). The most probable period for the onset of the first episode of major depression extends from mid-adolescence to mid-40s, but almost 40% experience their first episode of depression before age 20 years (2). Adolescent depression predicts several negative health outcomes in later life, including physical health problems, anxiety disorders and bipolar disorder (3). Besides, adolescent depression is associated with poor education attendance and increase risk of suicidal ideation (4). Thus, there is a strong clinical need for effective interventions in this area.

Selective serotonin reuptake inhibitors (SSRIs) are typically used as antidepressants in the treatment of adolescent depression. It has been widely acknowledged that it takes about at least 6–8 weeks for antidepressants to unfold their action entirely (5). According to the FDA, SSRIs can increase the risk of suicide in the first 2 weeks of treatment (6). Besides, it is still controversial that medications have side effects such as weight gain/loss, insomnia, and loss of appetite in treating adolescent depression (7). Therefore, there is a substantial unmet need for better and safer approaches for young people with depression.

For treatment-resistant major depressive disorder, HF-rTMS of the left dorsolateral prefrontal cortex (DLPFC) appears therapeutically effective, and low-frequency (LF) rTMS of the right DLPFC has similar efficacy. It uses electromagnetic induction to generate an electric current, stimulating the cortex and depression-related areas and altering dysfunctional brain patterns. There is a lot of peer-reviewed research examining the efficacy of rTMS (8, 9). A few studies suggest that it may better treat depression in adolescents compared to adults (10). Additionally, clinical guidelines recommend standard treatment protocol such as HF-rTMS applied to the left DLPFC for 4–6 weeks (5 daily treatments per week) (9). However, Hett et al. (11) find that the effects of 2 weeks rTMS are significant. One of the first blinded, randomized, controlled clinical study of the effectiveness of high-frequency TMS for treatment-resistant depression in adolescents find there is no statistically significant difference in the improvement of depressive symptom between the study group and sham group (12). Therefore, more systematic evidence is wanted to investigate the efficacy of rTMS in first-episode adolescent depression.

Therefore, this was a blinded, randomized clinical study that investigated the early effects of add-on rTMS in first-episode major depressive disorder, providing new ideas for treating adolescent depression. This study used the HAMD-17 and CDRS-R, THINC-it, and IVA-CPT to evaluate the clinical efficacy. We hypothesized that adolescents who received 10-Hz, left prefrontal TMS, delivered over the 10 sessions would have a more remarkable improvement in depressive symptoms and cold cognitive function than the control group.

## Methods and Design

Participants were recruited from in- and out-patients of the Hangzhou Seventh People’s Hospital. Inclusion criteria for patient selection were: (1) patients were between 12 and 18 years old; (2) males and females, Han nationality; (3) patients with DSM-IV clinical criteria for the major depressive episode; (4) patients who demonstrated a HAMD-17 score > 17 or CDRS-R score ≥ 40; and (5) patients who were medication-free and experiencing the first depressive episode.

Patients were excluded if they fell into any of the following standards: (1) left-handed; (2) suffered from a severe or unstable physical illness; (4) had a history of head injury or a seizure disorder; (5) were diagnosed with mental retardation, substance dependence or abuse (except nicotine and caffeine), bipolar disorder, obsessive-compulsive disorder, post-traumatic stress disorder, eating disorder defined by DSM-IV; (6) had metal implants; (7) took benzodiazepines within 12 h of cognitive assessments; (8) consumption of alcohol within 8 h of cognitive assessments.

The medical ethics committee approved this study in Hangzhou Seventh People’s Hospital in April 2021 (Ethical Research Review 2021, No. 32). It was conducted in accordance with the Code of Ethics of the World Medical Association (ChiCTR2100048534). All patients provided their written and informed consent and written informed consent were obtained from a parent or guardian for participants under 16 years old. Patients were randomly assigned to either the control group or the study group. For the control group, subjects took the sertraline for the first 2 weeks, while the study group received rTMS treatment on 10 consecutive workdays (Monday–Friday, for 2 weeks).

Both groups were given 50 mg sertraline daily for the first 2 weeks. During the following 2 weeks, both groups will continue with sertraline treatment without stimulation. Patients were administered 100 mg sertraline daily if the reduction of their HAMD-17 scores were below 50% within the first 2 weeks of treatment.

### Clinical Assessment

The severity of depression was assessed using HAMD-17 and CDRS-R at 0 (baseline), 2, and 4 weeks. The HAMD-17 was used as the primary outcome of measure and the CDRS-R as a secondary outcome. Similar to historical studies, we divided patients according to the reducing rate: (1) early improvers defined as a ≥ 20% reduction below baseline in HAMD-17 within the first 2 weeks (13); (2) responders were patients having a HAMD-17 score reduction of ≥ 50% below baseline. (3) remitters were patients having an HAMD-17 score reduction of ≤ 7 points below baseline.

### Neuropsychological Assessment

Neuropsychological tests were administered to assess cold executive function at baseline and 2 weeks after treatment, which were used as secondary outcome in the study. IVA-CPT assessed auditory and visual attention on the same task, which lasted about 20 min to complete instructions, warm-up, preliminary examination, and cool-down. Subjects were instructed to click the mouse when they saw or heard a “1” (target) and not click the mouse when they saw or heard a “2” (error). The response result of each stimulus was recorded on the computer for analysis. We recorded the Visual Attention Quotient, Auditory Attention Quotient, and Full-Scale Attention Quotient to evaluate the cold executive function’s inhibitory control and cognitive flexibility (14).

THINC-it was a brief screening tool designed to measure cognition and determined whether cognitive functioning was impaired. THINC-it included four objective cognitive tests (which were adapted from choice reaction time, 1-back working memory task, symbol digit coding, and Trails-B) and a subjective cognitive questionnaire (PDQ-5). McIntyre et al. (15) confirmed that THINC-it was a valuable and sensitive tool for detecting cognitive dysfunction for major depressive disorder (MDD). He recommended applying THINC-it to the measurement of all patients with MDD. Hou et al. (16) also validated the reliability and validity of the Chinese version of the THINC-it tool in MDD. Five indicators of THINC-it, including PDQ-5, Spotter, Symbol check, Codebreaker, and Trails, were recorded to evaluate subjects’ cold executive function.

### Stimulation Parameters

Magstim rapid stimulators with a figure-8 coil (Magstim, Sheffield, United Kingdom) were used by professional doctors. For every participant, the resting motor threshold (RMT) was determined before the initial rTMS session. RMT was defined as the lowest intensity able to elicit a visible muscle contraction of the right abductor pollicus brevis (APB) in five of 10 trials, with amplitude of at least 50 μV. The study group was stimulated with a frequency of 10 HZ, 90%RMT intensity for safety, 60 trains, 4 s on and 15 s off, 2,400 pulses per session, and five sessions per week. In each treatment session, stimulation was delivered to the left DLPFC, defined as the site 5-cm rule. The TMS treatments were assigned by a random number list. The patients were naive to rTMS prior to the study. Adverse events were recorded in TESS during and after each treatment session.

### Statistics

Statistical analyses were performed using SPSS Version 25.0. The measurement data conforming to the normal distribution were expressed as mean ± standard deviation. Pearson χ^2^-tests were used to investigate the differences between groups in the rates of early improvers at 2 weeks, responders at 2- and 4-week time points and remitters at 4 weeks. Changes in HAMD-17, CDRS-R scores, and neuropsychological assessments over time were analyzed with repeated-measures analysis of variance (ANOVA). *Post hoc* comparisons were performed with the paired *t*-test adjusted for multiple comparisons by the Bonferroni method. The differences between groups in changes from baseline, at 2 and 4 weeks, were analyzed with linear contrasts for the time multiplied by the rTMS interaction.

For the analysis of cognitive effects after DLPFC-rTMS, individual patients’ test scores on neuropsychological tests were first converted to z-scores. In the various tests of THINC-it, the score of PDQ5, Spotter and Trails were positively associated with the severity of the cognitive impairment. In terms of Symbol Check and Codebreaker, the original score was negatively related to the severity of the cognitive function. In the original THINC-it design, the direction of each test result should be consistent. PDQ5-D, Spotter, and Trails were multiplied by –1, so the results of five tests were positively associated with cold executive function (17). All statistical tests were two-tailed, and a *p*-value < 0.05 was considered to be significant.

## Results Demographics

The study recruited 100 patients, and only 97 subjects had completed all treatments. There were no significant changes in the blood routine, biochemistry, heart rate, blood pressure, electrocardiogram, and other results of the patients before and after treatment. Two patients (2%) in the study group chose to quit because of pain at the stimulation site. One patient (1%) in the control group replaced sertraline with fluoxetine due to the loss of appetite. Patients in the control group did not report any symptoms related to fatigue, and there were no patients who experienced mania during the study.

Forty-eight patients in the study group and forty-nine in the control group were administered 100 mg sertraline daily at the end of the second week because the reduction in their HAMD-17 scores was less than 50% compared to baseline. There were no differences in age, gender, education, the duration of the current episode, age at onset in the two groups. Relevant demographic and clinical characteristics of the two groups are shown in Table 1.

**TABLE 1:** Demographic and clinical characteristics of patients.

	Study group (*n* = 48)	Control group (*n* = 49)	*χ^2^/*t**	*P*
Gender (Female/male)	41/7	38/11	−0.055	0.956*^a^*
Age (years)	14.65 ± 2.34	15.39 ± 2.44	1.529	0.130*^b^*
Education (years)	10.79 ± 4.38	10.73 ± 2.94	−0.075	0.940*^b^*
Duration of current episode (months)	17.69 ± 14.08	15.55 ± 17.29	−0.667	0.507*^b^*
Age at onset (years)	13.35 ± 3.57	14.29 ± 2.55	1.481	0.142*^b^*

*Values are mean ± SD unless otherwise stated. ^a^Pearson χ^2^ test; ^b^Independent sample t-test.*

### Primary and Secondary Efficacy Outcome

There were no significant differences observed in the ratio of early improvers (who demonstrated a 20% reduction of HAMD-17 scores) between the two groups (95.83% vs 73.47%, χ^2^ = 9.277, *P* = 0.002) within the first 2 weeks of treatment. The responder rates (a 50% reduction of HAMD-17 scores) was significantly higher in the study group than the control group (45.83% vs 18.37%, χ^2^ = 8.412, *P* = 0.004) after 2 weeks and was significantly higher in the study group than the control group (62.50% vs 28.57%, χ^2^ = 11.262, *P* = 0.001) after 4 weeks. Remission of depression, which was evaluated at the end of the study, was found in 18 patients in the active group and in six patients in the control group. Difference was observed between the two groups regarding the ratio of remission (31.25% vs. 6.12%, χ^2^ = 10.130, *P* = 0.001).

**FIGURE 1:**
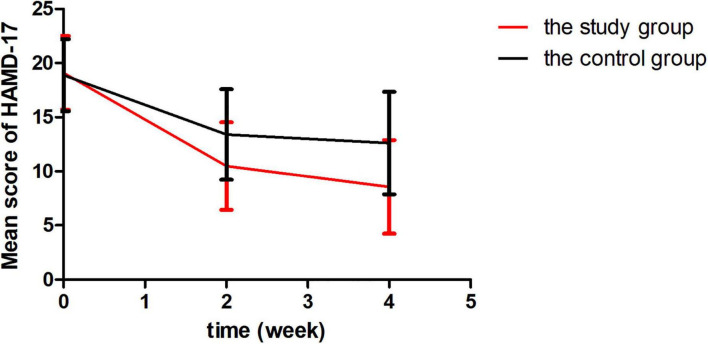
Reduction in Hamilton Depression rating scale 17 version (HAMD-17).

As shown in Table 2, there were no significant differences before the treatment between the two groups. However, the total score of HAMD-17 and CDRS-R were declined sharply than those before treatment. Time effects indicated that the score of HAMD-17 of two groups decreased more significantly as time went by (*F* = 192.86, *P* < 0.001; Figure 1). The time × group interaction indicated difference in symptom changed over time (*F* = 11.65, *P* < 0.001). *Post-hoc* comparison of HAMD-17 scores was performed. We observed no difference at the baseline (*P* > 0.05), but a significant difference was found at the 2-week time point (at the end of rTMS; *P* < 0.01) that remained significant until the fourth week of treatment (*P* < 0.01).

**TABLE 2:** The effects of time and group variables on a total score on HAMD-17and CDRS-R.

	Study group (*n* = 48)	Control group (*n* = 49)	*P*	Time	Groups	Time × groups
HAMD				*F* = 192.86 *P* = 0.000	*F* = 12.91 *P* = 0.001	*F* = 11.65 *P* = 0.000
0w	19.10 ± 3.42	18.88 ± 3.33	0.742			
2w	10.48 ± 4.06	13.41 ± 4.19	0.001			
4w	8.56 ± 4.32	12.61 ± 4.74	<0.001			
CDRS-R				*F* = 44.89 *P* = 0.000	*F* = 10.21 *P* = 0.002	*F* = 21.13 *P* = 0.000
0w	76.90 ± 19.03	73.88 ± 13.41	0.370			
2w	54.92 ± 18.71	67.67 ± 20.06	0.002			
4w	49.17 ± 19.96	69.65 ± 20.33	<0.001			

*Values are mean ± SD; with repeated-measures analysis of variance. This is a 2-week double-blind study with a 2-week extended antidepressant phase.*

**FIGURE 2:**
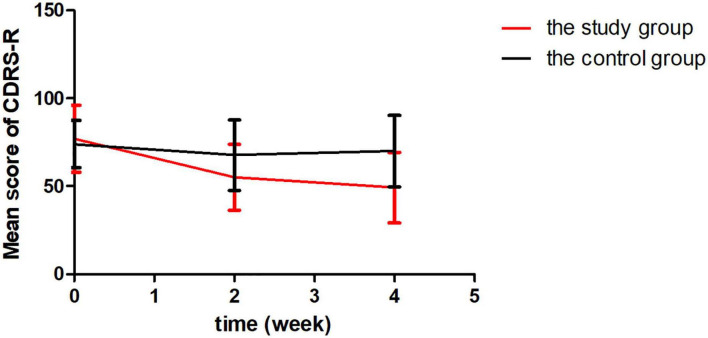
Reduction in Children’s Depression Rating Scale-Revised (CDRS-R).

Consistently with the score of HAMD-17, the CDRS-R score improved over time (*F* = 44.89, *P* < 0.001; Figure 2). There was a significant difference between the two groups, with a lower CDRS-R score in the study group compared with the control group after two weeks (*P* < 0.01) and four weeks (*P* < 0.001). In addition, no significant differences in HAMD-17 or CDRS-R scores were found between the two groups at baseline.

Baseline neuropsychological assessment did not differ between the two groups. The neuropsychological test score is summarized in Table 3.

**TABLE 3:** Neuropsychological rating of patients.

CPT/THINC-it	Study group (*n* = 48)	Control group (*n* = 49)
Attention Quotient (listening) baseline	0.06 ± 0.97	−0.06 ± 1.04
2w	0.35 ± 0.79*^ac^*	−0.34 ± 1.07*^a^*
Attention Quotient (visual) baseline	0.07 ± 0.94	−0.07 ± 1.06
2w	0.25 ± 0.85^*b*^	−0.24 ± 1.08
Full Scale Attention Quotient baseline	0.10 ± 0.92	−0.10 ± 1.07
2w	0.32 ± 0.88*^ac^*	−0.31 ± 1.02*^a^*
PDQ-5 baseline	−0.07 ± 1.02	0.07 ± 0.99
2w	0.18 ± 0.95	−0.18 ± 1.03
Spotter baseline	−0.10 ± 0.89	−0.10 ± 1.10
2w	0.21 ± 0.73^*b*^	−0.21 ± 1.18
Symbol Check baseline	0.05 ± 0.95	−0.05 ± 1.05
2w	−0.003 ± 0.98	0.003 ± 1.03
Code breaker baseline	0.01 ± 1.02	−0.01 ± 0.99
2w	0.08 ± 0.87	−0.08 ± 1.12
Trails baseline	0.32 ± 0.84*^b^*	−0.31 ± 1.05
2w	−0.01 ± 0.95	0.01 ± 1.05*^a^*

*Values are mean ± SD. ^a^P < 0.05 relative to baseline; ^b^P < 0.05, ^c^P < 0.01 between groups; with repeated-measures analysis of variance.*

Before treatment, there was no statistical difference in the Attention Quotient (listening), Attention Quotient (visual), and Full-Scale Attention Quotient of IVA-CPT between the two groups. After treatment, Attention Quotient (listening), Attention Quotient (visible), and Full-Scale Attention Quotient in the study group were significantly higher than those of the control group (*P* < 0.05).

As for THINC-it, there was no significant difference in PDQ-5, Spotter, Symbol Check, Codebreaker, and Trails during the study and did not differ between the two groups. However, we found that the study group showed improvement in Spotter compared to the control group (*t* = −2.121, *P* = 0.037).

## Discussion

This is the most extensive blinded, randomized clinical study to date examining the efficacy of 10-Hz add-on rTMS for first-onset adolescent depression. This study demonstrated that the total scores of HAMD-17 and CDRS-R in the study group were significantly lower than those in the control group after 2 weeks of treatment. We confirmed that left high-frequency rTMS combined with sertraline could improve the depressive symptoms of first-onset adolescent depression. According to the recent meta-analysis, there were only two randomized controlled trials (RCTs) compared the efficacy and tolerability of rTMS plus antidepressant and antidepressant alone in the treatment of first-episode depression (18). The response to rTMS in our study echoed the study of first-episode depression with 2 weeks period of either rTMS or sham rTMS (10 HZ, 90% motor threshold) (19), which reported early improvers (a 20% reduction of HAMD-17) are significantly different (57% vs. 29%) at the first 2 weeks. Another study of first-episode depressed participants who had received paroxetine treatment (20–40 mg per day) in combination with a 4-week period of either rTMS or sham rTMS of the DLPFC (10 Hz, 80% motor threshold), indicated that rTMS at 10-Hz accelerated the onset of action and augmented the response to paroxetine (20). Zhang et al. (10) compared 42 adolescents with 75 adults and find a response rate of 94.1% and a remission rate of 88.2% among adolescents. Similarly, MacMaster et al. (21) treated 32 adolescents with treatment-resistant depression with left-side high-frequency rTMS and suggested that 56% achieved response (HAMD-17 reduction rate ≥ 50%), 44% achieved remission (HAMD-17 ≤ 7 points).

However, the present findings were inconsistent with other previous studies. The study by Croarkin et al. (12) involved 103 adolescent patients diagnosed with treatment-resistant depression who received monotherapy TMS (active or sham) about 3,000 pulses per day for a 6-week trial. No difference in efficacy was found between the active TMS monotherapy (*n* = 48) or sham TMS (*n* = 55). The contrasting results may be related to the sample size and the severity of depression as well as the placebo response of TMS masked the true effects of TMS. Similarly, other randomized controlled trials showed that adjunctive HF-rTMS of the DLPFC was not more effective than sham rTMS in the treatment of depression (22, 23). The common characteristics of these studies are that the patients had previous depressive episodes (>3), diagnosed with treatment-resistant depression or much older. These factors likely contributed to the lack of efficacy of adjunctive rTMS treatment for depression.

What’s more, neuropsychological assessments such as IVA-CPT and THINC-it showed that the cold executive function of the two groups improved after treatment. The IVA-CPT results confirmed that the study group’s cold executive function was significantly improved compared with the control group. Consistent with the results of previous studies, a meta-analysis (24) found that high-frequency rTMS stimulations on DLPFC enhance TMT performance for depression. TMT is responsible for visual scanning and movement, processing speed, and working memory. After 77 depression patients received 30 courses of high-frequency rTMS treatment on DLPFC, Collier et al. (8) found that rTMS could improve the ability of inhibitory control for depression. Our study expanded the sample size and used THINC-it and IVA-CPT to evaluate the cold executive function, adding to the growing body of literature that suggested wilder clinical application of rTMS for adolescent depression.

Structural neuroimaging showed decrease in total gray matter PFC volume starting at around 11–12 years old (25) due to synaptic pruning. Zheng et al. (26) showed that rTMS could enhance axon regeneration ability and up-regulate the brain-derived neurotrophic factors (BDNF), accounting for the progress of cognitive function. To et al. (27) found that stimulated L-DLPFC could change the central executive network (CEN) and produced a wide range of network effects and linked with other default networks. The study to explore the molecular mechanisms of the fronto-limbic circuit of patients with MDD found that 10-Hz left PFC rTMS modulated left PFC glutamate levels and decreased left PFC P60 in MDD patients (28). Glutamate is the primary excitatory neurotransmitter, with roles in neurogenesis, synaptogenesis, neuronal migration, cognition, learning, and memory. The treatment time of rTMS and the total amount of stimulation is also closely related to the efficacy. Hett et al. (11) pointed out that the most commonly used protocol of rTMS treatment was high frequency (10 HZ, 120% MT). About 30 courses (6–8 weeks) of treatment significantly improve clinical symptoms, but it is time-consuming and costly. Dhami et al. (29)reported that after 2 weeks of 10 courses of θ burst magnetic stimulation (TBS), there was a statistically significant difference in the improvement of clinical symptoms for adolescent depression (*P* < 0.05). Zhang et al. (10) expanded the sample size and found that 10 courses of high-frequency rTMS therapy combined with antidepressants had a synergistic effect in adolescent depression. This study verified that high-frequency rTMS for 2 weeks of 10 courses had an early add-on effect on first-episode adolescent depression and improved patients’ clinical symptoms as well as the cold executive function.

However, this study still has a few considerable impediments. Firstly, the control group did not take the sham TMS treatment because of the limit of time and foundation. We will continue to do the placebo group study in the future. The other limitation was the placebo effect, and previous studies demonstrated that more study sites and lower baseline depressive symptom severity were positively associated with high placebo effect (30, 31). We restricted the number of study sites and recruited patients with the major depressive disorder to address the problem. Thirdly, there was a limitation relates to the lack of evaluating the long-term efficacy because of the high drop-out rates after hospitalization. Since lack of SSRI effects at 2 weeks does not mean SSRIs are less effective because at least 6–8 weeks is need to assess efficacy of SSRIs. Fourth, our study did not include imaging biomarkers or other biological assessments that provide more convincing evidence of neural mechanisms that underlined cognitive effects.

## Conclusion

In conclusion, our results support that high-frequency rTMS can improve depressive symptoms and cold executive function of first-onset adolescent depression. Further studies will expand the sample size and integrate imaging biomarkers or have a longer follow-up to explore the effect of rTMS combined with sertraline on first-episode adolescent depression.

## Data Availability Statement

The original contributions presented in this study are included in the article/supplementary material, further inquiries can be directed to the corresponding author/s.

## Ethics Statement

The studies involving human participants were reviewed and approved by the medical ethics committee approved this study in Hangzhou Seventh People’s Hospital in April 2021 (Ethical Research Review 2021, No. 32). Written informed consent to participate in this study was provided by the participants’ legal guardian/next of kin.

## Author Contributions

HC and JG conceptualized the study and funding proposal, as well as drafted the manuscript. XH revised the manuscript and provided expert consultation on the design of the study. HH, XW, and CX analyzed the data. All authors critically reviewed the manuscript and approved of it in its final form.

## Conflict of Interest

The authors declare that the research was conducted in the absence of any commercial or financial relationships that could be construed as a potential conflict of interest.

## Publisher’s Note

All claims expressed in this article are solely those of the authors and do not necessarily represent those of their affiliated organizations, or those of the publisher, the editors and the reviewers. Any product that may be evaluated in this article, or claim that may be made by its manufacturer, is not guaranteed or endorsed by the publisher.

## Abbreviations

HF-rTMS: high-frequency rTMS
LF-rTMS: low-frequency rTMS
DLPFC: left dorsolateral prefrontal cortex
HAMD-17: Hamilton depression rating scale 17 version
CDRS-R: Children’s Depression Rating Scale-Revised
IVA-CPT: Integrated visual and auditory continuous performance test
SSRIs: Selective serotonin reuptake inhibitors
FDA: Food and Drug Administration
SC: Symbol check
RMT: resting motor threshold
APB: abductor pollicus brevis
ANOVA: repeated-measures analysis of variance
TMT: Trail Making Test
BDNF: brain-derived neurotrophic factors
CEN: central executive network
TBS: burst magnetic stimulation.
